# DNA methylation signatures of sporadic colorectal cancer with microsatellite instability

**DOI:** 10.1080/15592294.2026.2711191

**Published:** 2026-08-10

**Authors:** Rebecca Ward, Molly Endicott, Bethan Mallabar-Rimmer, Joe Burrage, Kitty Sherwood, Qiwen Huang, Joseph C. Ward, Steve Thorn, Connor Woolley, Sophie J. Wood, Emma Dempster, Harry D. Green, Ian Tomlinson, Amy P. Webster

**Affiliations:** a Department of Clinical and Biomedical Sciences, Faculty of Health and Life Sciences, University of Exeter, Exeter, UK; b Institute of Genetics and Cancer, University of Edinburgh, Edinburgh, UK; c Department of Oncology, University of Oxford, Oxford, UK

**Keywords:** DNA methylation, colorectal cancer, mismatch repair, CIMP

## Abstract

Colorectal cancer (CRC) is a heterogeneous disease shaped by genetic and epigenetic alterations. Approximately 20% of CRCs exhibit widespread CpG island hypermethylation, termed the CpG Island Methylator Phenotype (CIMP), frequently accompanied by *MLH1* promoter hypermethylation, deficient mismatch repair (dMMR) and microsatellite instability (MSI). However, methylation patterns associated with MSI, independent of CIMP and *MLH1* silencing, and the influence of anatomical location and patient age on the CRC methylome remain incompletely defined. We performed epigenome-wide DNA methylation profiling of 259 sporadic CRCs using the Illumina EPICv2 array. Differential methylation between MSI and microsatellite stable (MSS) CRCs was assessed after adjustment for tumour purity and anatomical location, then *MLH1* promoter methylation and CIMP status, to delineate MSI-associated methylation changes. Additionally, we evaluated the effects of anatomical location and age on methylation patterns. While differential methylation between MSS and MSI CRCs was dominated by *MLH1* promoter hypermethylation, additional adjustment for *MLH1* hypermethylation and CIMP identified 656 CpG sites associated with MSI, beyond the global methylator phenotype. These included hypermethylation at *LRP6*, *GSK3β*, and *CDK12*, identifying differential methylation of genes involved in WNT signalling and transcriptional regulation. Within MSI CRCs, we observed the co-occurrence of *MLH1* hypermethylation with promoter hypermethylation at *TXNRD1*. Anatomical location was strongly associated with methylation, whereas age had more modest effects. These results identify methylation changes associated with sporadic MSI beyond CIMP status and *MLH1* hypermethylation, reveal heterogeneity within MSI CRCs, and indicate that anatomical location is a major determinant of the CRC methylome, advancing molecular stratification of CRC.

## Introduction

Colorectal cancer (CRC) is the second leading cause of cancer-related mortality worldwide [1]. In addition to genetic drivers, epigenetic dysregulation is a defining molecular feature of CRC that shapes tumour initiation and evolution [2,3]. DNA methylation, the addition of a methyl group to cytosine-phosphate-guanine (CpG) dinucleotides, is a key epigenetic mechanism regulating gene expression and is frequently disrupted in cancer [4].

Approximately 20% of CRCs display concordant hypermethylation at CpG islands against a background of global hypomethylation, a feature termed the CpG Island Methylator Phenotype (CIMP) [5,6]. CIMP-high (CIMP-H) tumours are associated with distinct clinicopathological features including *BRAF* mutation, older age and proximal colon location [7]. A common downstream consequence of CIMP is epigenetic silencing of *MLH1* through promoter hypermethylation, leading to deficient mismatch repair (dMMR) and microsatellite instability (MSI) [8]. While CIMP-driven *MLH1* hypermethylation represents the most common mechanism of dMMR in sporadic CRC, a subset of tumours develop dMMR through biallelic somatic mutations in MMR genes in the absence of a global methylator phenotype.

Since CIMP, *MLH1* promoter hypermethylation and MSI are highly correlated molecular features, most methylation changes observed in sporadic MSI CRC are thought to reflect upstream CIMP-driven CpG island hypermethylation. However, it remains unclear whether *MLH1* silencing or MSI are associated with additional, more specific methylation alterations independent of the broader methylator phenotype. Distinguishing methylation changes attributable to CIMP or *MLH1* silencing from those that accompany MSI independently is therefore necessary to determine the extent to which MSI contributes to epigenetic heterogeneity in CRC. A more refined molecular characterisation of methylation changes associated with CIMP, *MLH1* silencing and MSI may improve understanding of inter-tumour variability in tumour biology and immunotherapy response, and advance precision oncology approaches in CRC [9].

We hypothesised that MSI may be associated with methylation patterns that are partially distinct from CIMP. To explore this, we sought to (1) characterise methylation differences associated with MSI after accounting for CIMP status and *MLH1* promoter hypermethylation, (2) isolate methylation changes associated with *MLH1* silencing independent of the global methylator phenotype, and (3) evaluate the influence of anatomical location and patient age on the CRC methylation landscape.

## Methods

### Ethics approval

Genomic and phenotypic data used in this study were obtained as part of the 100,000 Genomes Project (100kGP) study from the National Genomic Research Library (Genomics England), a research resource with approval to operate as a Research Tissue Bank by the HRA/Cambridge Central Research Ethics Committee (REC reference 20/EE/0035), with the principles of the Helsinki Declaration embedded into day-to-day operations. Data from the National Genomic Research Library can be accessed from within the secure Genomics England Research Environment, subject to institutional agreements [10]. Normal tissue samples were collected in accordance with ethical guidelines approved by the South Central (Oxford B) Research Ethics Committee (REC reference 05/Q1605/66) and the HRA/Cambridge Central Research Ethics Committee (REC reference 20/EE/0035).

### Study cohort and clinical data

**Figure 1.**
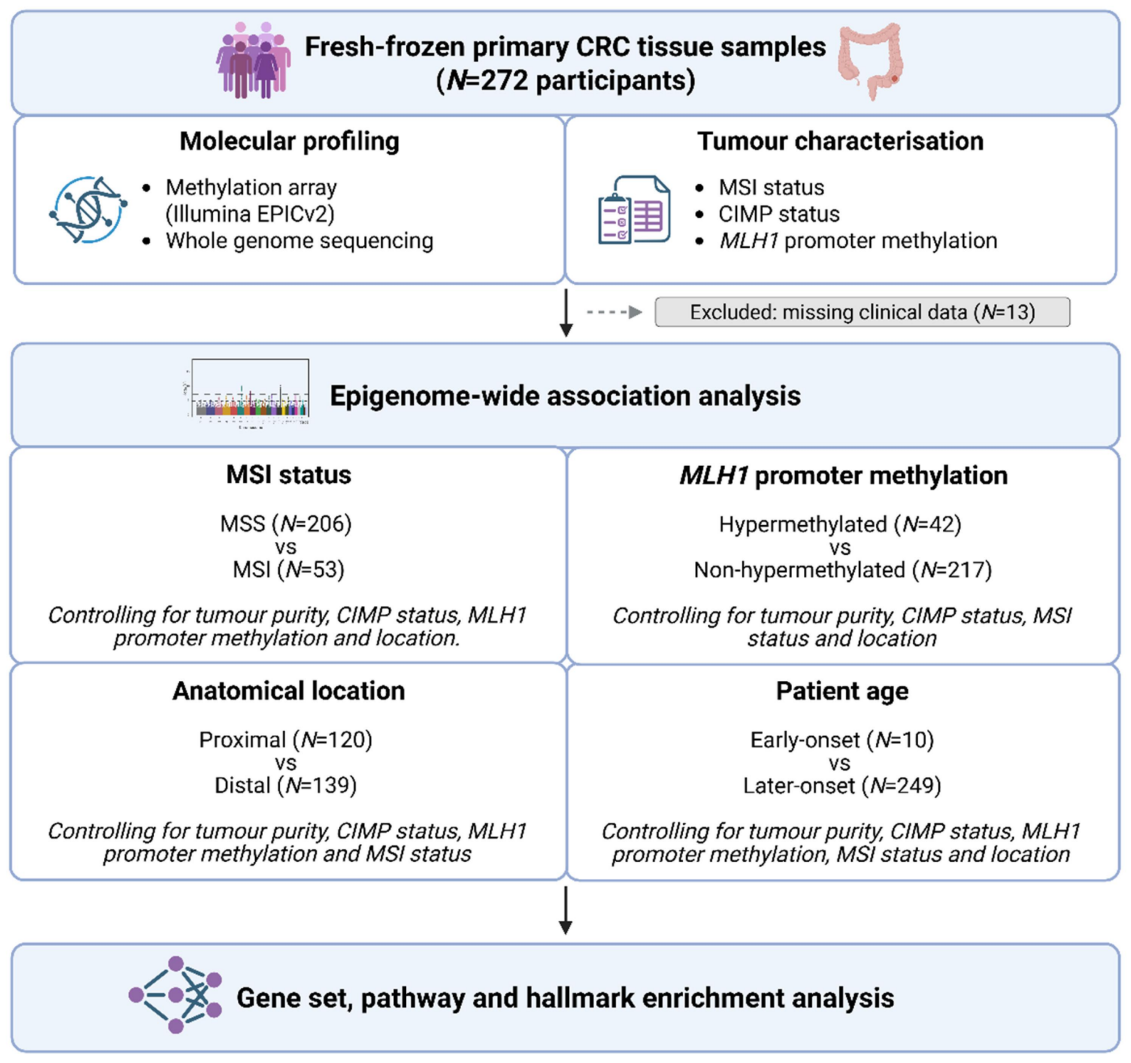
Study design. Flow diagram showing sample inclusion criteria and overview of the epigenome-wide association analyses performed. CRC, colorectal cancer; CIMP, CpG island methylator phenotype; MSS, microsatellite stable; MSI, microsatellite instability.

The study cohort comprised 272 primary fresh-frozen CRC tissue samples and 5 normal tissue controls. Whole-genome sequencing (WGS) and clinical data, including patient sex, age at sampling and tumour anatomical location were obtained through the Genomics England (GEL) Research Environment (RE) as part of the 100kGP main programme v18 release. Participants with Lynch syndrome were identified by the presence of a pathogenic germline variant in an MMR gene (*MLH1*, *MSH2*, *MSH6*, *PMS2*) using WGS data and were excluded from the study cohort. Tumour anatomical location information was derived from the av_tumour table within the RE and categorised as proximal (right-sided), including the caecum, ascending colon, hepatic flexure, and transverse colon; distal (left-sided), including the splenic flexure, descending colon, sigmoid colon; or rectal, including the rectosigmoid junction and rectum. For EWAS analysis, anatomical location was dichotomised into proximal and distal (including rectal). Microsatellite instability (MSI) status was determined using Detecting Microsatellite Instability by Next-Generation Sequencing (mSINGS) [11], run with default settings on tumour WGS data aligned to GRCh38 within the RE. Samples with missing MSI status or anatomical location data were excluded, resulting in a final cohort of 259 CRC and 5 normal tissue samples. A flow diagram of the study design and sample inclusion criteria is shown in Figure 1.

### DNA methylation data generation

DNA methylation data was generated from fresh-frozen tissue samples using the Illumina Infinium MethylationEPIC v2.0 BeadChip, which measures methylation at 937,691 CpG sites across the genome. For each sample, 500ng DNA was bisulphite-converted using the EZ DNA methylation-Gold kit (Zymo Research), according to the manufacturer’s protocol. To minimise batch effects, samples were randomised across bisulphite conversion plates, BeadChips and array positions.

### Data preprocessing and quality control

Data preprocessing, including sample-level and CpG site-level quality control was performed according to the following previously published pipeline (Hannon et al. [12]; https://github.com/ejh243/BrainFANS). Briefly, raw array IDAT files were imported into R using the bigmelon package (version 1.31.1) [13] and quality control checks were conducted using the wateRmelon package (version 2.11.4) [14]. Poor quality samples were excluded using the following criteria; (i) median signal intensity of <500, (ii) median bisulphite conversion efficiency <80%, (iii) >1% probes with detection *p* > 0.05, (iv) samples with missing data for >2% of probes, (v) normalised violence <0.1 (wateRmelon qual()) function, (vi) sample identity mismatches based on 65 SNP targeting probes. Poor quality probes were excluded using the following criteria; (i) detection *p* > 0.05 in more than 1% of samples, (ii) probes with common SNPs (minor allele frequency >5%) in the single base extension position based on those identified by Lehne et al. [15], (iii) mis-mapped or mapping to non-specific regions.

Methylation levels at each CpG were defined as beta-values (β-values), which were calculated as the ratio of methylated signal intensity to the sum of the methylated and unmethylated signal intensity at each probe. To correct for technical variation between probes, and ensure comparability across samples, raw signal intensities were normalised using the Dasen function within the wateRmelon package.

### Tumour purity estimation

Tumour purity was estimated from the normalised β-values using InfiniumPurify (version 1.3.1) [16]. Tumour cell fraction was inferred with the getPurity() function using informative differentially methylated CpG sites (iDMCs) derived from TCGA colon adenocarcinoma as the reference dataset by specifying tumour.type = ‘COAD.’

### Classification of *MLH1* promoter methylation

We selected four CpG probes, located within the *MLH1* promoter, based on the critical regulatory region defined by Deng et al [17]. To classify promoter hypermethylation, we adapted the method described by Benhamida et al. for the Infinium MethylationEPIC v1.0 array [18]. Three of the four probes (cg23658326, cg21490561, and cg00893636) used are present on both v1.0 and v2.0 arrays and were validated by Benhamida et al. The fourth probe, cg19132762, is specific to the v2.0 array and was included because of its proximity to the critical promoter region (22 bp upstream). Methylation thresholds for each probe were calculated as the mean β-value of normal tissue controls plus three standard deviations. Samples were classified as *MLH1-*hypermethylated if the β-value at each of the four probes exceeded the methylation threshold. Samples with β-values below the threshold for any of the four probes were classified as non-*MLH1-*hypermethylated. Threshold values for each probe are provided in Supplementary Table S1.

### Classification of CIMP status

CIMP status was defined using the recursively-partitioned mixture model (RPMM) clustering method applied in previous CRC methylation studies [19–21]. Briefly, RPMM clustering (RPMM R package, version 1.25) was performed on the 10% most variable autosomal probes (*n* = 84,886), resulting in CRCs being assigned into one of four methylation clusters, CIMP-High, CIMP-Low, Cluster 3 and Cluster 4, with Cluster 3 and Cluster 4 collectively described as ‘Non-CIMP.’

### Principal components analysis

Principal Component Analysis (PCA) was performed on the normalised β-values using the prcomp() function in R. To account for differences in DNA methylation across the genome, β-values for each CpG site (variable) were centred to a mean of zero and scaled to unit variance (Z-score transformation), prior to singular value decomposition. The proportion of variance explained by each principal component (PC) was evaluated using a scree plot (Supplementary Figure S1). Pearson correlation coefficients were computed between PCs and known clinical or technical covariates to identify potential confounders for inclusion in downstream regression models.

### Differential methylation analysis

Differentially methylated CpG sites were identified using the limma R package (version 3.64.1) [22], which fits a linear model for each CpG site to test the association between methylation and the variable of interest. The models were adjusted for potential confounders identified through PCA. Limma applies empirical Bayes moderation to the standard errors, which enhances statistical power by borrowing information across all probes, yielding more stable and reliable parameter estimates [23]. Untransformed β-values were used as input to the model, with the estimated effect sizes corresponding directly to Δβ (difference in mean β-values) to facilitate interpretability. While β-values can violate linear regression assumptions of residual normality at the extremes of the distribution of methylation values, empirical simulations have demonstrated that this does not introduce systemic false-positive bias in EWAS analyses [24]. Significance was defined using a Bonferroni-adjusted *p*-value threshold of *p* < 5.91×10^−8^ (to account for 846,923 individual probe-level tests).

### Genomic annotation of CpG sites

CpG sites were annotated to genes using a modified version of the Infinium MethylationEPICv2.0 manifest, re-annotated based on the GENCODE Human Release 47 (GENCODE v47) reference database [25]. The genomic location of CpG sites relative to CpG islands was also classified as; CpG Islands (CGI), Shore (within 0–2 kb of a CpG island), Shelf (within 2–4 kb of a CpG island) and non-CGI-related sites.

### Gene set and pathway enrichment analysis

To determine whether significantly differentially methylated CpG sites were enriched in specific biological pathways, functional enrichment analysis was performed using the gometh() function within the *missMethyl* R package (version 1.42.0) [26]. This method accounts for probe-density bias due to differences in CpG representation across genes on the Illumina EPICv2 array. Enrichment was tested using the Gene Ontology (GO) and Kyoto Encyclopaedia of Genes and Genomes (KEGG) pathway databases. GO terms and KEGG pathways were considered significantly enriched at a False Discovery Rate (FDR) <0.05.

### Analysis of promoter methylation at CRC driver genes

To investigate methylation changes with potential functional impact on gene regulation in CRC, we performed a focused analysis on promoter regions of CRC driver genes. A curated list of 82 CRC driver genes was obtained from the Integrative OncoGenomics (IntOGen) database (version 2024.09.20) [27], which identifies driver genes by aggregating and analysing somatic mutation data across multiple cancer types. Promoter-associated CpG sites were defined as those annotated as within 200 base pairs upstream of the transcriptional start site (TSS200), using GENCODEv47. Differential methylation analysis was performed on the subset of promoter-associated CpG sites in CRC driver genes using limma as described previously. A Bonferroni-adjusted *p*-value threshold of *p* < 1.04 × 10^−4^ was applied to account for 479 probes.

### Data handling and statistical analysis

Methylation data preprocessing and quality control were performed using R (v4.2.1), functional enrichment analysis was conducted using R (v4.5), all other analyses were performed using R (v4.3.3). Summary statistics for cohort clinical variables were generated separately for MSS and MSI groups (Table 1). Categorical variables were reported as counts and percentages, and between-group differences were assessed using Fisher’s exact test. Continuous variables were summarised as mean ± standard deviation and compared using Welch’s *t*-test. For methylation analyses, Bonferroni correction was applied to adjust for multiple testing in the limma regression models. All other statistical tests were two-sided, and a *p*-value < 0.05 was considered statistically significant.

**Table 1.** Summary of the study cohort clinical and molecular characteristics. Counts ≤ 5 have been masked to preserve participant confidentiality. Comparisons between MSI and MSS groups were performed using Fisher’s exact tests for categorical variables and Welch’s two-sample *t*-test for continuous variables.

Characteristic	MSS	MSI	*P* value
Total (N)	206	53	
Age (mean ± SD)	69.5 ± 10.4	70.2 ± 11.7	*p* = 0.67
Sex			*p* = 3.59 × 10^−5^
Female	67 (32.5%)	34 (64.2%)	
Male	139 (67.5%)	19 (35.8%)	
Anatomical location			*p* = 7.31 × 10^−12^
Proximal colon	73 (35.4%)	47 (88.7%)	
Distal colon	76 (36.9%)	≤5	
Rectal	57 (27.7%)	≤5	
*MLH1* promoter status			*p* = 7.2 × 10^−31^
Hypermethylated	≤5	39 (73.6%)	
Non-hypermethylated	203 (98.5%)	14 (26.4%)	
CIMP status			*p* = 1.96 × 10^−19^
CIMP-High	18 (8.7%)	38 (71.7%)	
CIMP-Low	44 (21.4%)	≤5	
Non-CIMP	144 (69.9%)	11 (20.8%)	
Stage			*p* = 0.436
I	28 (13.6%)	≤5	
II	74 (35.9%)	25 (47.2%)	
III	57 (29.4%)	16 (30.2%)	
IV	14 (6.8%)	≤5	
Unknown	33 (16%)	≤5	

CIMP, CpG island methylator phenotype; MSI, microsatellite unstable; MSS, microsatellite stable; SD, standard deviation.

## Results

### Cohort characteristics and principal determinants of methylation variance

**Figure 2.**
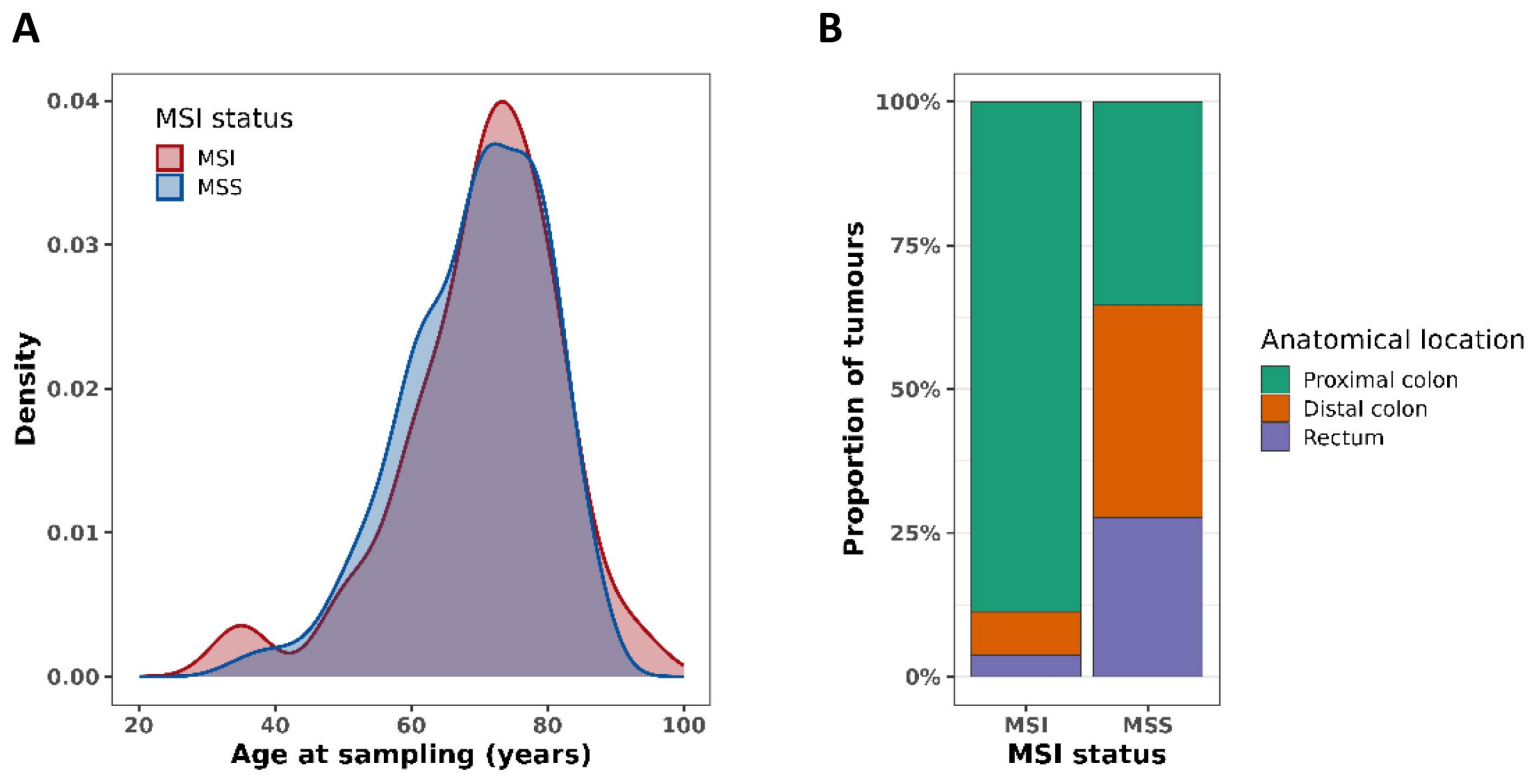
Clinical characteristics of colorectal cancer cohort stratified by microsatellite instability status (*n* = 206 MSS; *n* = 53 MSI). (A) Density plot showing the age distribution at sampling for MSI and MSS samples. (B) Stacked bar chart showing relative proportions of MSI and MSS samples across anatomical locations of the colorectum. MSS, microsatellite stable; MSI, microsatellite instability.

We analysed epigenome-wide methylation and whole-genome sequencing data from 259 primary CRC cases, comprising 206 MSS and 53 MSI tumours (Figure 1). Clinical and molecular characteristics are summarised in Table 1. The non-*MLH1*-hypermethylated MSI CRC consisted of cases with confirmed biallelic somatic MMR alterations, monoallelic pathogenic somatic variants where a second hit had not been identified and cases where no definitive somatic MMR variant was identified (Supplementary Table S2). Consistent with previous studies, MSI CRC was more frequent in female patients (64.2% vs 32.5%, *p* = 3.59×10^−5^; Fisher’s exact test) and showed a strong association with anatomical location (*p* = 7.31×10^−12^; Fisher’s exact test), with 88.7% of MSI CRCs arising in the proximal colon compared with 35.4% of MSS CRCs (Figure 2B). As expected, MSI status was significantly associated with both *MLH1* promoter hypermethylation (73.6% of MSI vs < 5% of MSS CRC, *p* = 7.2×10^−31^; Fisher’s exact test) and CIMP status (*p* = 1.96×10^−19^; Fisher’s exact test), with 71.7% of MSI CRC classified as CIMP-High compared to 8.7% of MSS CRC.

We performed PCA on normalised β-values to identify technical and biological sources of methylation variance. Correlation of principal components (PCs) with clinical and molecular features revealed that MSI status, tumour purity, anatomical location, and *MLH1* promoter methylation were strongly associated with the first two PCs, each explaining > 5% of the total variance (Supplementary Figures S1-S2). To account for potential confounding, these variables were included as covariates in downstream regression models when not the primary variable of interest. PCA score plots (PC1–PC2) coloured by key variables showed clean separation of MSI and MSS CRC along the first component, consistent with widespread methylation reprogramming associated with dMMR (Supplementary Figure S3).

### Epigenome-wide methylation differences between MSS and MSI CRC

**Figure 3.**
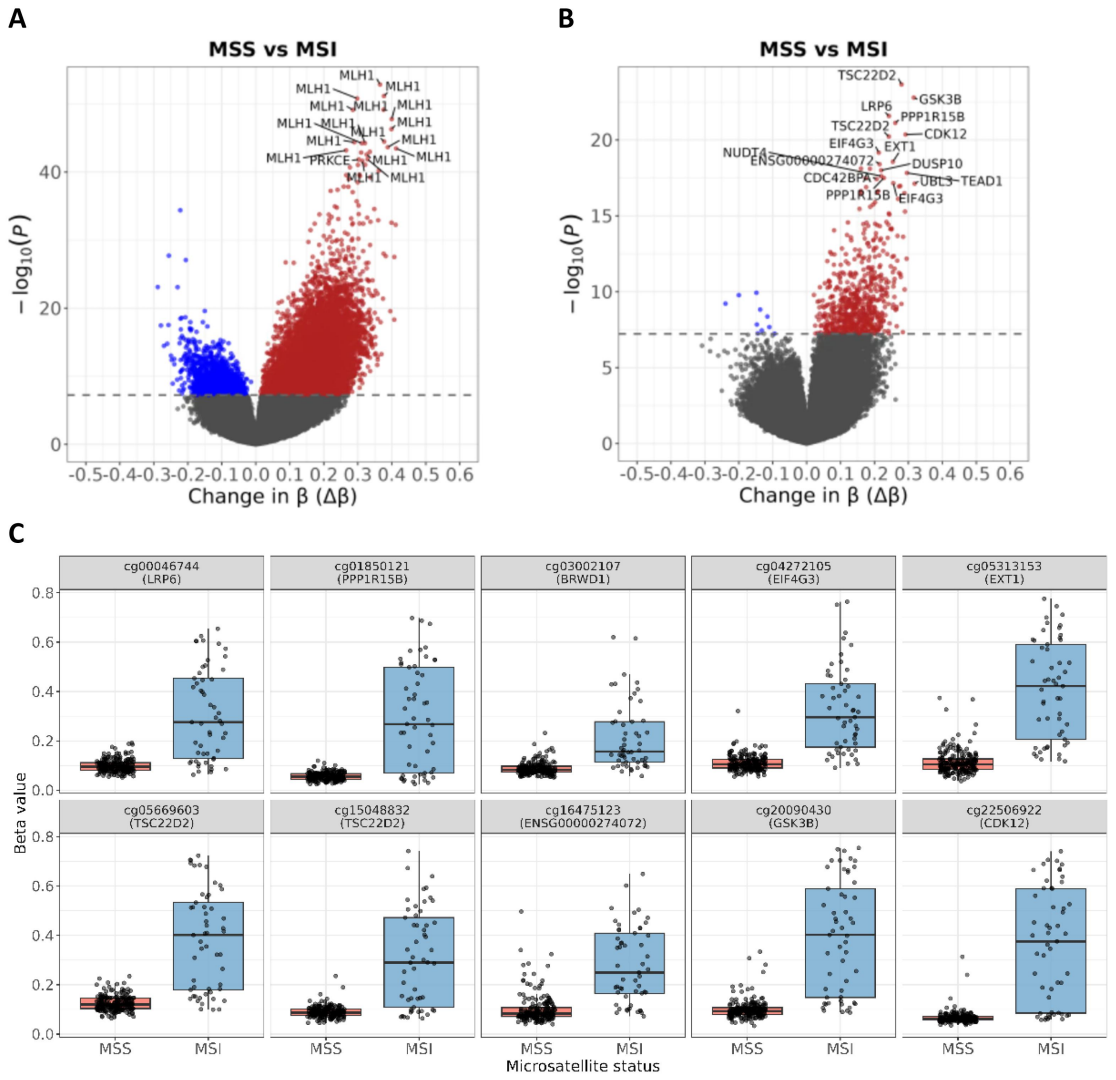
Epigenome-wide association study of DNA methylation in microsatellite stable (MSS; *n* = 206) and microsatellite unstable (MSI; *n* = 53) colorectal cancer. Volcano plots showing the association between methylation (difference in average beta, Δβ) and statistical significance (−log_10_ *p*-value) for (A) MSS vs MSI adjusted for tumour purity and anatomical location only, and (B) MSS vs MSI adjusted for tumour purity, anatomical location, *MLH1* promoter methylation and CIMP status. Each point represents a CpG site, top differentially methylated CpG site with Δβ > 0.15 are labelled with gene annotations. (C) Boxplots showing beta-values for the top 10 significantly differentially methylated CpG sites between MSS and MSI tumours, after adjustment for tumour purity, anatomical location, *MLH1* promoter methylation and CIMP status. These CpG sites represent the strongest epigenetic differences independent of the CIMP/*MLH1*-hypermethylated pathway and highlight potential methylation markers of mismatch repair deficiency. CIMP, CpG island methylator phenotype. MSS, microsatellite stable; MSI, microsatellite instability.

An initial epigenome-wide association study (EWAS) comparing MSI (*n* = 53) and MSS (*n* = 206) CRC, without controlling for CIMP or *MLH1* promoter methylation status, identified 22,975 significantly differentially methylated positions (DMPs) (Bonferroni-corrected threshold *p* < 5.91×10^−8^, inflation λ = 4.11), confirming extensive global methylation differences between MSS and MSI CRCs (Supplementary Figure S4 and Supplementary Table S3). As anticipated, the signal was dominated by CpG sites at the *MLH1* promoter locus (Figure 3A). In addition to DMPs located in the *MLH1* promoter, we also observed other notable hypermethylated loci, including four consecutive significant DMPs annotated to *PRKCE* (median Δβ = 0.31, IQR: 0.29–0.33), highlighting additional loci that may be involved in the broader methylation changes characteristic of *MLH1*-hypermethylated MSI CRC.

### Methylation differences associated with MSI after accounting for CIMP and MLH1 silencing

To disentangle the independent contribution of MSI from effects of *MLH1*-hypermethylation or CIMP, we performed a subsequent analysis adjusting for both CIMP status (categorised as CIMP-H and CIMP-Low/negative) and *MLH1* promoter methylation in addition to tumour purity and anatomical location. We identified a substantially smaller set of 656 significant DMPs (*p* < 5.91 × 10^−8^, Supplementary Table S4) and a marked reduction in test statistic inflation (λ = 1.37 vs 4.11), representing methylation differences that are independent of the broader methylator phenotype (Supplementary Figure S5).

The majority (96.9%) of significant DMPs were hypermethylated in MSI relative to MSS CRC (Figure 3B). Of the most significantly associated DMPs, a prominent subset mapped to genes involved in WNT signalling including *LRP6*, a WNT co-receptor; *GSK3β*, a regulator of β-catenin stability; and *EXT1*, a heparan-sulphate polymerase involved in ligand presentation (Figure 3C). Further notable hypermethylated DMPs annotated to genes involved in TGF-β signalling (including *TSC22D2*, *TEAD1* and *SMAD3*) and genes related to transcriptional or RNA regulatory processes (*CDK12*, *EIF4G3* and *PPP1R15B*). To determine whether these MSI-associated loci converge on specific biological processes, we performed functional enrichment analysis of significant DMPs using Gene Ontology (GO) and Kyoto Encyclopaedia of Genes and Genomes (KEGG) databases. No GO terms or KEGG pathways reached statistical significance after multiple-testing correction (FDR < 0.05, Supplementary Table S5), suggesting that methylation changes associated with MSI are not clustered within canonical pathways.

Together, these findings demonstrate that, after accounting for the effects of the CIMP and *MLH1* promoter hypermethylation, only a limited residual set of methylation differences distinguishes MSS and MSI subtypes. The marked attenuation of both the number of significant loci and test statistic inflation after adjustment indicates that the majority of methylation differences between MSI and MSS CRC reflect upstream CIMP-related processes, with only a modest residual signal associated with MSI status.

### Methylation differences associated with MLH1 promoter hypermethylation after accounting for CIMP

*MLH1* promoter hypermethylation, arising as a downstream consequence of CIMP, is the primary epigenetic mechanism leading to sporadic MSI in CRC. However, *MLH1* promoter hypermethylation may also function as a distinct epigenetic event associated with methylation changes in addition to those of the broader methylator phenotype. We therefore sought to characterise methylation patterns accompanying *MLH1* promoter hypermethylation by comparing *MLH1*-hypermethylated and non-*MLH1*-hypermethylated tumours, adjusting for tumour purity, anatomical location, and CIMP status. This analysis identified 3,508 significant DMPs (*p* < 5.91 × 10^−8^, inflation λ = 1.33, Supplementary Table S6), reflecting widespread background hypermethylation characteristic of CIMP (Supplementary Figure S6). As expected, the most significant associations mapped to the *MLH1* promoter region. Several additional loci showed concurrent hypermethylation in *MLH1*-hypermethylated tumours, including *B3GALT1* and *SCNNB1* (*p* < 5.91 × 10^−8^). Gene set and pathway enrichment analysis of *MLH1*-associated differentially methylated positions (DMPs) highlighted genes involved in developmental and neurogenic processes, as well as ion channel activity (FDR < 0.05, Supplementary Figure S7A and Supplementary Table S7). Enriched pathways included neuroactive ligand – receptor interactions, neuroactive ligand signalling and calcium signalling (Supplementary Figure S7B), suggesting that *MLH1*-associated methylation changes may influence cell signalling beyond canonical DNA repair pathways.

### Co-methylation of MLH1 with TXNRD1 in MSI CRC

**Figure 4.**
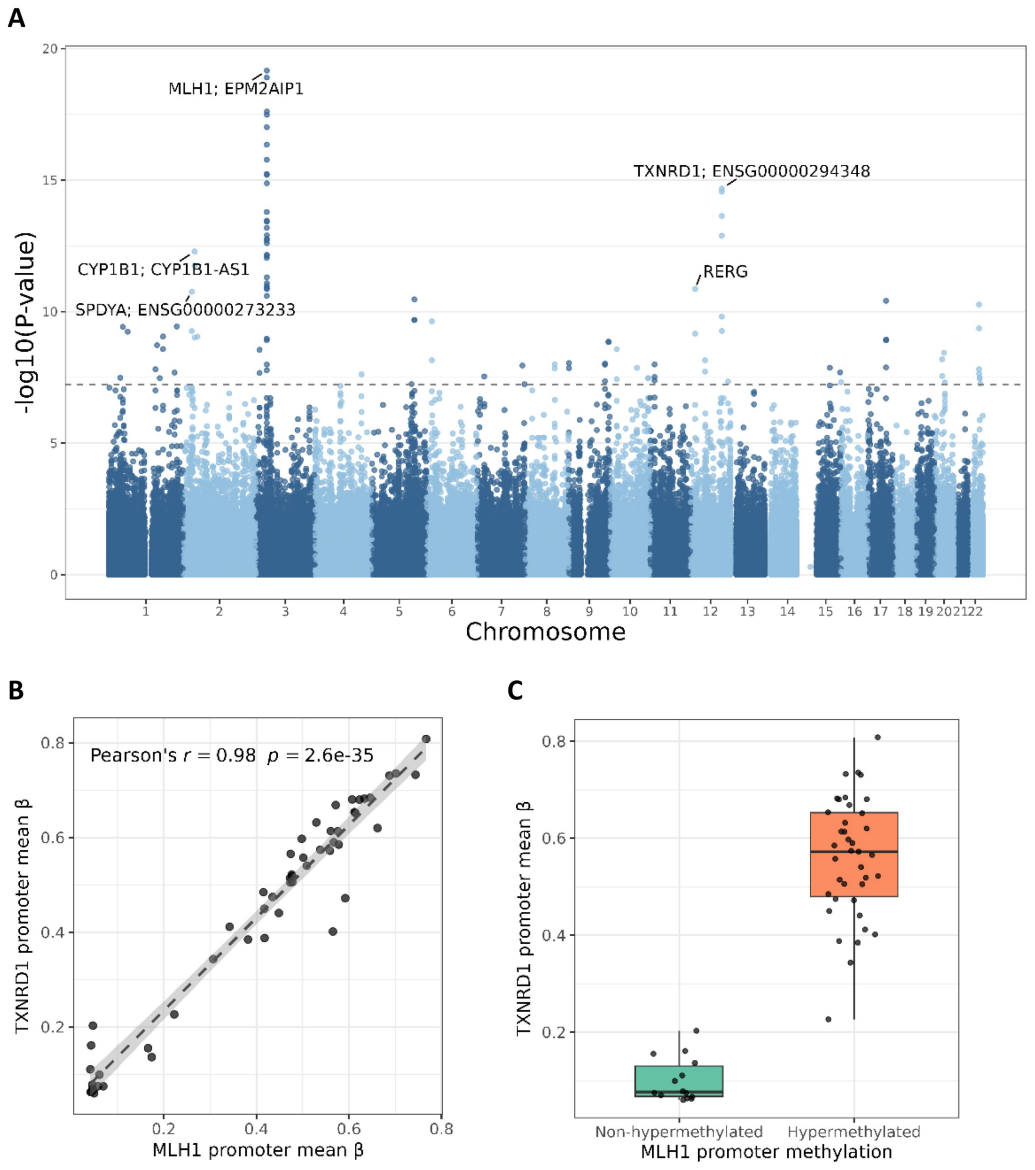
Epigenome-wide association of microsatellite unstable (MSI) colorectal cancer (CRC) with (*n* = 39) and without (*n* = 14) *MLH1* promoter hypermethylation, adjusting for tumour purity, anatomical location and CIMP status. (A) Manhattan plot showing −log_10_ *p*-values for all CpG sites, Bonferroni-corrected epigenome-wide significance (*p* < 5.91 × 10^−8^) is indicated by the grey dashed line. (B, C) Scatter plots showing the relationship between mean promoter methylation (CpG sites annotated to TSS200) in *MLH1* and *TXNRD1*. Dashed lines represent linear regression fits, with correlation (r) calculated using Pearson’s correlation. (C) *TXNRD1* promoter methylation by *MLH1* promoter hypermethylation status. Boxplots showing beta values for TSS200 CpGs in *TXNRD1* across *MLH1*-hypermethylated versus non-hypermethylated MSI CRC.

To distinguish methylation changes driven by *MLH1* silencing from those arising through alternative MSI mechanisms, we directly compared *MLH1*-hypermethylated (*n* = 39) and non-*MLH1*-hypermethylated (*n* = 14) MSI tumours, adjusting for tumour purity, anatomical location and CIMP status (Figure 4A). We identified 81 significant DMPs mapping to 29 unique genes (*p* < 5.91 × 10^−8^; λ = 0.97, Supplementary Table S8). In addition to the *MLH1* locus, top DMPs were annotated to *TXNRD1*, a thioredoxin reductase enzyme involved in the regulation of cellular oxidative stress, *CYP1B1*, and *RERG*. Five hypermethylated CpG sites were located within the *TXNRD1* promoter, and *MLH1* promoter methylation was strongly correlated with *TXNRD1* promoter methylation (Pearson’s *r* = 0.98, *p* < 8.1 × 10^−37^, Figures 4B,C), consistent with co-occurring promoter hypermethylation in *MLH1*-hypermethylated MSI. Although significant DMPs were distributed across multiple genes, enrichment analyses did not yield any significantly enriched GO terms or KEGG pathways at an FDR < 0.05 (Supplementary Table S9).

### Impact of anatomical location and patient age on DNA methylation profiles

**Figure 5.**
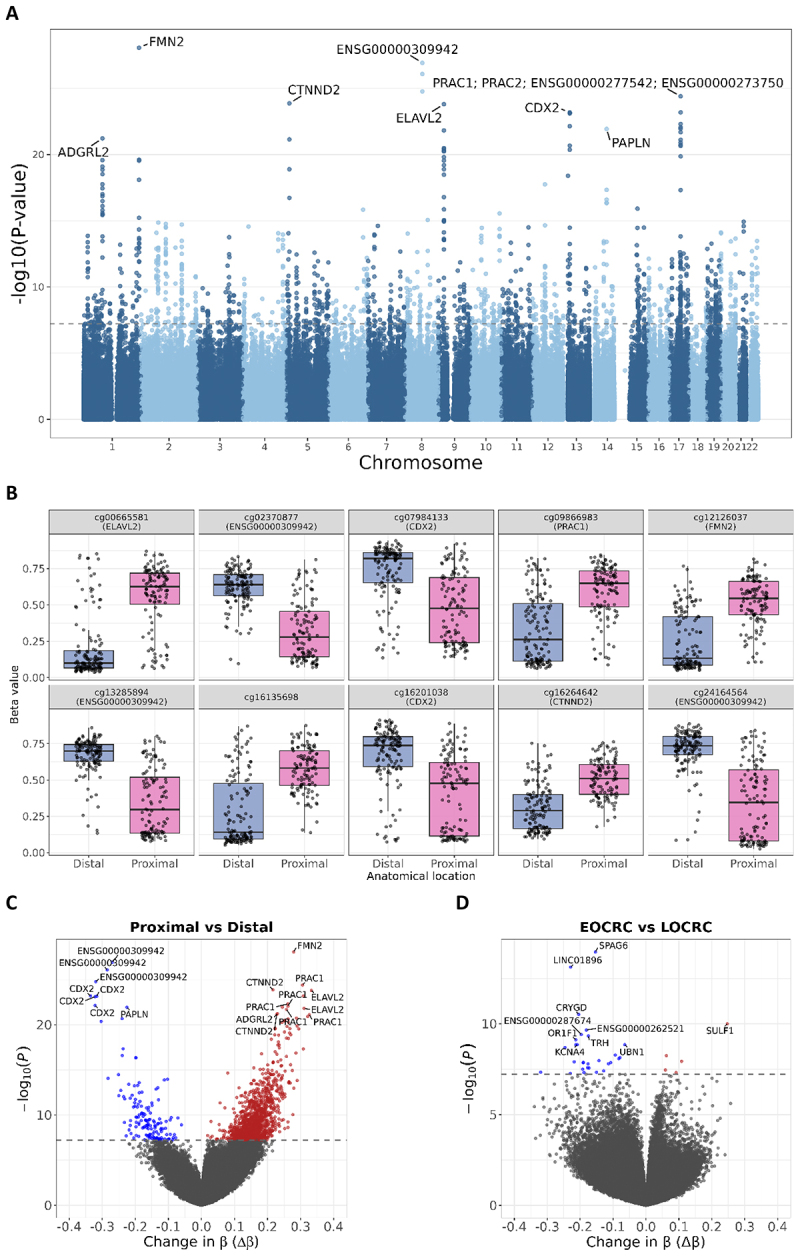
Epigenome-wide association analysis of anatomical location and patient age in colorectal cancer (CRC). (A) Manhattan plot showing −log_10_ *p*-values for differentially methylated CpG sites between proximal (*n* = 120) and distal (*n* = 139) CRC, adjusted for tumour purity, MSI status, CIMP status, and *MLH1* promoter hypermethylation. The grey dashed lineindicates the Bonferroni-corrected epigenome-wide significance threshold (*p* < 5.91 × 10^−8^). (B) Boxplots showing the distribution of β-values for the top 10 significantly differentially methylated CpG sites between proximal and distal CRC. (C) Volcano plot showing methylation differences (Δβ) against statistical significance (−log_10_ *p*-value) for proximal vs distal CRC. (D) Volcano plot showing methylation differences for early-onset CRC (EOCRC, *n* = 10) vs later-onset CRC (LOCRC, *n* = 249). Genes annotated to the most significant differentially methylated CpG sites are labelled. MSI, microsatellite instability; CIMP, CpG island methylator phenotype.

PCA showed that anatomical location was a major source of variation in global CRC methylation profiles. We next assessed methylation differences by anatomical location, comparing proximal (*n* = 120), and distal (*n* = 139) CRCs, adjusting for tumour purity, CIMP status, *MLH1* promoter methylation and MSI status. We identified 1,428 significant DMPs (*p* < 5.91 × 10^−8^, inflation λ = 2.14, Supplementary Figure S8A), annotated to 727 unique genes, indicating marked differences in methylation with anatomical location (Figure 4A, Supplementary Table S10). The majority (89.8%) of significant DMPs were hypermethylated in proximal CRC. Among the most significant DMPs were CpG sites annotated to *CDX2*, a homeobox intestinal transcription factor critical for intestinal differentiation; *CTNND2*, a known oncogenic driver; *FMN2*, a putative tumour suppressor; and *PRAC1*, *PDE10A* and *ELAVL2* (Figure 5B). Proximal CRCs showed coordinated hypomethylation of five consecutive CpG sites in the *CDX2* promoter region (TSS1500), and 12 adjacent CpG sites within the promoter of *PDE10A*. In contrast, *FMN2*, *PRAC1/2* and *ELAVL2* showed consistent hypermethylation across multiple promoter-associated CpG sites (Figure 5C). These results demonstrate that anatomical location is a major determinant of CRC methylation patterns, independent of MSI or CIMP status and *MLH1* promoter methylation.

Gene ontology analysis of significant DMPs revealed strong enrichment for developmental and morphogenetic processes, consistent with the distinct embryonic origins of the proximal (midgut) and distal (hindgut) colon (Supplementary Figure S9 and Supplementary Table S11). Top enriched terms included multicellular organism development, system development, and anatomical structure morphogenesis (FDR < 0.05). Enriched molecular function categories highlighted regulators of chromatin and transcription, including DNA-binding transcription factor activity and RNA polymerase II cis-regulatory region binding, indicating preferential involvement of promoter- and enhancer-associated regulatory regions.

We next evaluated the contribution of patient age to methylation variation. Using age as a continuous variable and adjusting for MSI status, tumour purity, anatomical location, CIMP status, and *MLH1* promoter methylation, we did not identify any significant DMPs at the Bonferroni-adjusted threshold (*p* < 5.91 × 10^−8^, inflation λ = 0.92, Supplementary Figure S10 and Supplementary Table S12). In contrast, when modelling age as a binary variable comparing early-onset CRC (EOCRC, <50 years, *n* = 10) and later-onset CRC (LOCRC, ≥50 years, *n* = 249), we identified 34 significant DMPs (Supplementary Table S13). The most significant loci mapped to genes including *SPAG6*, *LINC01896*, *SULF1*, and *CRYGD* (Figure 5D).

### Analysis of promoter methylation at CRC driver genes

To investigate whether age or anatomical location influences the epigenetic regulation of known CRC driver genes, we performed a targeted analysis of 82 driver genes curated from the Integrative OncoGenomics (IntOGen) database, specifically focusing on CpG sites within the promoter regions (TSS200). Restricting differential methylation analysis to these regions, while controlling for tumour purity, CIMP status, *MLH1* promoter methylation and MSI, revealed 4 DMPs significantly associated with anatomical location after Bonferroni correction (*p* < 1.04 × 10^−4^). Of the significantly associated CpG sites, 3 were located within the promoter region of *GRIN2A* and 1 within the promoter region of *ERBB2*, all of which were hypermethylated in proximal compared distal tumours (Supplementary Figure S11A, Supplementary Table S14). In contrast, no CpG sites within these driver gene promoters were significantly associated with patient age (Supplementary Figure S11B, Supplementary Table S15). These results suggest that specific epigenetic divergence in *GRIN2A* and *ERBB2* may reflect distinct oncogenic pathways between proximal and distal CRC, whereas patient age has limited impact on driver gene promoter methylation.

## Discussion

In this study, we investigated whether methylation differences associated with MSI could be identified after accounting for the overlapping effects of *MLH1* promoter hypermethylation and the broader CIMP-related changes. We identified a subset of methylation differences associated with MSI that remained after adjustment for these correlated molecular features, supporting the hypothesis that the methylation landscape of MSI CRC is not fully explained by CIMP-associated hypermethylation alone. We also identified methylation differences associated with *MLH1* promoter hypermethylation, anatomical location and, to a lesser extent, patient age. Together, these findings refine the molecular characterisation of CRC subtypes and improve our understanding of the epigenetic factors contributing to methylation heterogeneity in CRC.

Consistent with its established role as the primary driver of sporadic MSI, an initial comparison of MSI and MSS CRC revealed extensive methylation differences dominated by hypermethylation at the *MLH1* promoter and widespread CIMP-associated changes. In addition to *MLH1* hypermethylation, we also observed significant hypermethylation at other genes, including *PRKCE*, a serine-threonine kinase reported to stabilise TET1 via protein phosphorylation [28]. Although the high genomic inflation observed in this analysis warrants cautious interpretation, given the role of TET1 in active demethylation and its association with CIMP [29], *PRKCE* hypermethylation may represent a CIMP-related feature contributing to altered TET1 regulation.

After controlling for *MLH1* promoter methylation and CIMP status we identified a distinct set of DMPs associated with sporadic MSI. The most significantly associated loci mapped to genes involved in WNT signalling, including *GSK3β*, *LRP6* and *EXT1* as well as regulators of transcription control such as *CDK12*, *TSC22D2* and *EIF4G3*. *GSK3β* promotes β-catenin degradation in the cytoplasmic destruction complex, *LRP6* functions as a cell surface co-receptor initiating ligand-dependent activation and *EXT1*, a heparan-sulphate polymerase required for WNT and FGF ligand presentation [30,31]. While aberrant WNT activation in CRC is typically driven by *APC* mutations, these findings identify differential methylation of genes involved in WNT signalling in MSI CRC, although GO terms and KEGG pathway over-representation analyses were not significant after FDR correction. Functional validation is required to determine whether these methylation differences directly influence WNT activity.

Additionally, *CDK12* (cyclin-dependent kinase 12) regulates transcription of DNA damage repair genes [32]. Loss or reduced expression of *CDK12* through promoter hypermethylation may contribute to genomic instability in non-*MLH1*-hypermethylated MSI CRC. Interestingly, hypermethylation at *TSC22D2*, encoding Transforming Growth Factor β-Stimulated Clone 22 Domain Family Member 2, has recently been reported as a robust biomarker for MSI detection and is positively associated with features predictive of immunotherapy response, including tumour mutational burden, neoantigen load, and expression of immune checkpoint molecules [33]. Our independent identification of this locus provides further support for *TSC22D2* methylation as a clinically relevant epigenetic marker of MSI.

Direct comparison of *MLH1*-hypermethylated and non-*MLH1*-hypermethylated MSI CRC revealed a small set of co-methylated loci, most notably including *TXNRD1*. *TXNRD1* encodes a thioredoxin reductase enzyme involved in mitigating the accumulation of reactive oxygen species (ROS) [34], suggesting a potential link between altered methylation patterns and oxidative stress pathways in MSI CRC. The strong correlation between *TXNRD1* and *MLH1* promoter hypermethylation suggests coordinated epigenetic regulation within *MLH1*-hypermethylated MSI CRC. This raises the possibility that impaired oxidative stress responses could represent a recurrent feature associated with the *MLH1*-hypermethylated MSI subgroup. Although our analysis adjusted for CIMP status, *MLH1* promoter methylation and CIMP are closely related biological processes, that may not be able to be completely disentangled statistically. Therefore, these findings should be interpreted as methylation changes associated with the *MLH1*-hypermethylated MSI subgroup rather than effects uniquely attributable to *MLH1* promoter hypermethylation. Nonetheless, the consistent co-occurrence of *TXNRD1* and *MLH1* promoter hypermethylation identifies *TXNRD1* as a potentially functionally relevant locus that warrants further investigation.

Consistent with previous studies reporting transcriptomic and epigenetic differences between proximal and distal CRC [35–38], anatomical location emerged as a substantial source of DNA methylation variation in our cohort, after adjustment for MSI status, CIMP status, *MLH1*-hypermethylation, and tumour purity. Gene ontology analysis of significant DMPs demonstrated overrepresentation of developmental and morphogenetic processes, consistent with previous studies profiling methylation in proximal and distal CRC [36]. These findings suggest that genes involved in developmental regulation are enriched in methylation differences associated with location, although whether this reflects distinct embryological origins, tumour dedifferentiation, or broader epigenetic reprogramming during carcinogenesis remains to be determined.

Among the most significant loci, were CpG sites annotated to *CDX2, ELAVL2, PRAC1/2* and *PDE10A*. CDX2 is an intestinal transcription factor and tumour suppressor that maintains colonic epithelial differentiation and limits proliferation, partly through repression of Wnt/β-catenin signalling [39]. We observed hypomethylation of CpG sites annotated to *CDX2* in proximal compared with distal CRC, consistent with previous observations both in adenomas and adenocarcinomas [36,37]. Reduced CDX2 expression in proximal CRC has been associated with dMMR, CIMP-H and poor prognosis [40–42]. Our findings extend these observations by demonstrating differential methylation at the *CDX2* locus between proximal and distal CRC after adjustment for key molecular confounders.

Conversely, we observed hypermethylation at multiple CpG sites at *PRAC1/2* and *ELAVL2* in proximal CRC, aligning with previously reported reduced expression in proximal CRC [36,38,43,44] and supporting location-specific epigenetic regulation of this gene. Additionally, the differential methylation of *PDE10A*, which has been shown to be selectively upregulated in colorectal tumour tissue compared to normal mucosa [45], is of interest given its emergence as a potential therapeutic target [46].

In contrast, we observed only a modest impact of patient age at diagnosis on DNA methylation patterns in our cohort, suggesting that the contribution of chronological age to methylation variation was modest relative to anatomical location and tumour-associated molecular alterations. When age was modelled as a continuous variable, no CpG sites reached epigenome-wide significance after multiple testing correction, suggesting that age-related methylation effects are not a dominant feature of the CRC methylome. Although age has been associated with both methylation changes in normal tissues [47,48], and with *MLH1* hypermethylation in CIMP-H CRC (Levine et al. [48]), our PCA and EWAS results suggest that age-related methylation changes may be less pronounced in tumour tissue relative to other tumour-associated epigenetic alterations. When age was instead modelled as a binary variable to compare EOCRC (age < 50 years) and LOCRC (age ≥ 50 years), we identified 34 significant DMPs, including loci annotated to *SPAG6*, *LINC01896*, *SULF1*, and *CRYGD*. *SULF1* has recently been shown to be highly expressed in cancer-associated fibroblasts [49]. These findings potentially reveal methylation differences reflecting distinct biological processes between EOCRC and LOCRC. However, the small size of the EOCRC group limits statistical power and independent validation in larger cohorts is required.

Through targeted analysis of promoter methylation in established CRC driver genes, we identified hypermethylation of three loci within the *GRIN2A* promoter in proximal CRC. Since our analysis controlled for CIMP status, *MLH1* promoter methylation, and MSI status, these results indicate that *GRIN2A* hypermethylation may be independent of the global CpG hypermethylation typically observed with CIMP. *GRIN2A* is a tumour suppressor, and its dysregulation has been previously linked to increased tumour recurrence [50]. While further functional validation would be required to confirm the impact on gene expression, these findings suggest that epigenetic silencing of *GRIN2A* may contribute to the distinct clinical trajectories observed in proximal CRC. In contrast, the absence of significant associations with patient age suggests that promoter methylation of these 82 CRC driver genes is not significantly influenced by chronological age in this cohort.

We acknowledge several limitations of this study. Firstly, the use of bulk tumour tissue precluded resolution of cell-type-specific methylation patterns. Although we adjusted regression models for tumour purity, this approach does not capture variations in cellular composition, such as differences in immune infiltration, which are known to vary between MSS and MSI subtypes and represent a common source of residual confounding in EWAS analyses [51]. As a result, some of the observed methylation signals associated with MSI may partially reflect differences in immune or stromal cell proportions rather than tumour-intrinsic changes. Single-cell methylation profiling would offer higher-resolution insights into the cell-type specificity of the methylation changes we observed. Secondly, absence of matched normal tissue, limited interpretation of the tumour-specificity of methylation differences.

An additional limitation relates to the comparison of *MLH1*-hypermethylated and non-*MLH1*-hypermethylated MSI CRC. Although adjustment for CIMP status was performed to minimise the influence of the global methylator phenotype, MLH1 promoter hypermethylation is closely linked to CIMP-H and these molecular features cannot be completely disentangled statistically. Consequently, some residual confounding is likely to remain. Furthermore, although germline Lynch syndrome cases were excluded, the non-*MLH1*-hypermethylated MSI subgroup is biologically heterogeneous. While this group included cases with biallelic somatic MMR variants, we were unable to definitively identify the underlying mechanism of MSI in all cases. Therefore, the observed differences should be interpreted as methylation patterns associated with *MLH1*-hypermethylated and non-*MLH1*-hypermethylated MSI CRC, rather than as effects uniquely attributable to *MLH1* promoter hypermethylation. Future studies with more refined molecular subclassification, such as well-characterised hereditary and biallelic somatic dMMR cohorts, will be essential to disentangle universal MSI features from the epigenetic patterns associated with specific MSI subtypes. Additionally, we did not account for prior treatments (e.g., chemotherapy, radiotherapy) and other exposures like smoking history, which are known to influence DNA methylation, and could introduce residual confounding [52]. Finally, while we have identified differential methylation signals, the functional impact of these differences on gene expression was not examined. Future studies integrating matched normal tissue, gene expression data (e.g., RNA-seq), and independent validation cohorts will be important to confirm the clinical relevance of the methylation changes identified.

## Conclusions

Here we define a distinct DNA methylation landscape specifically associated with sporadic MSI in CRC, after statistical adjustment for *MLH1* promoter hypermethylation and CIMP status. The most significant loci mapped to genes with established roles in WNT signalling and transcriptional regulation, including *LRP6*, *GSK3β*, *CDK12*, and *TSC22D2*, identifying candidate epigenetic alterations associated with MSI CRC. We further identify co-occurrence of promoter hypermethylation of *MLH1* and *TXNRD1* in MSI CRC, suggesting a novel link between MSI and cellular oxidative stress regulation.

In addition to MMR status, we demonstrate that anatomical location is a major independent determinant of the CRC methylome, with proximal and distal CRC displaying distinct methylation profiles across developmental and regulatory genes including *CDX2*, *PRAC1/2*, and *PDE10A*. In contrast, we find only modest effects of patient age at diagnosis on methylation variation after adjustment for molecular and clinical covariates. Together, these findings refine the epigenetic classification of CRC, highlight novel candidate markers for molecular stratification, and provide a foundation for functional studies linking methylation patterns to CRC biology and therapeutic response.

## Supplementary Material

Supplementary_Figures.docx

## Data Availability

Data from the National Genomic Research Library (NGRL) used in this research are available within the secure Genomics England Research Environment. Access to NGRL data is restricted to adhere to consent requirements and protect participant privacy. Data used in this research include; clinical and genomic data from the 100kGP Main Programme Data Release v18. Clinical data including patient age, sex and tumour anatomical location was extracted using the cancer_analysis and av_tumour tables. The full list of Genomics England participant IDs together with clinical information and scripts can be found at ‘re_gecip/cancer_colorectal/rward/Ward_et_al.2026_CRC_methylation.’ Access to NGRL data is provided to approved researchers who are members of the Genomics England Research Network, subject to institutional access agreements and research project approval under participant-led governance. For more information on data access, visit: https://www.genomicsengland.co.uk/research. The supplementary tables and figures generated during this study are available in the Figshare repository: https://doi.org/10.6084/m9.figshare.32159106.
